# Freedom dreaming to STEM: A conceptual model for Black youth’s racial and STEM identity development through social media

**DOI:** 10.3389/fpsyg.2022.944207

**Published:** 2022-12-08

**Authors:** Tate LeBlanc, Aerika Brittian Loyd

**Affiliations:** Department of Psychology, University of California, Riverside, Riverside, CA, United States

**Keywords:** identity development, racial identity, STEM, social media, Black, youth

## Abstract

Social media use has become increasingly enmeshed in the lives of youth. Although investigations in this area have tended to focus on risk (e.g., cyberbullying) and negative outcomes (e.g., in mental health and academic functioning), a growing body of literature suggests there may be positive developmental outcomes associated with Black youths’ social media use. Social media may offer Black youth a means of resisting negative experiences, expand their opportunities to create and explore, and facilitate the integration of their racial and STEM identities. Aligned with PYD perspectives and PVEST, we suggest this dynamic process occurs iteratively within youth and bidirectionally between youth and their environment (online and offline) over the course of development. In this article, we present a conceptual model to guide future investigations to address gaps in the literature and elucidate the linkages between social media use, racial identity, and STEM identity among Black youth. We begin by reviewing two frameworks that provide the theoretical foundations for our model. We then discuss our outcomes of interest: racial identity and STEM identity. Given its rapidly evolving nature, we then proceed with a discussion about how social media may be operationalized, noting limitations in the current literature and highlighting the unique ways in which social media differs from offline contexts. Subsequently, we present our conceptual model, which we situate within distal, proximal, and individual offline influences. We also propose processes that may link social media use to positive identity outcomes. We conclude this article with recommendations for future investigations.

## Introduction

Social media use has become increasingly enmeshed in the lives of youth. Converging studies suggest that the majority of youth (i.e., adolescents and emerging adults ages 13–24) access social media platforms (e.g., Facebook, Instagram, and Twitter) several times each day (Hill et al., 2016; Villanti et al., 2017; Anderson and Jiang, 2018; Twenge et al., 2019; Auxier and Anderson, 2022). Further, Black youth, particularly adolescents, use social media at higher rates compared to their peers of other racial groups (Lenhart et al., 2015; Stevens et al., 2019). Over the past 2 years alone, social media has taken on increased importance for Black youth as a mode of support seeking (Parker et al., 2021) and social activism (Baskin-Sommers et al., 2021) in light of the ongoing dual pandemics of COVID-19 and anti-Black racism (Jones, 2021). Indeed, compared to their other-race and older counterparts, Black youth face unique racism-related (e.g., racial discrimination; Seaton, 2020) and age-related (e.g., restricted access to resources; DeJong and Love, 2015) stressors. As Black youths’ social media use becomes increasingly prominent amidst these unique stressors, examining individual differences in their social media use as it relates to their developmental trajectories remains paramount.

Although investigations in this area have tended to focus on risk (e.g., cyberbullying; Edwards et al., 2016) and negative outcomes (e.g., in mental health and academic functioning; Rose and Tynes, 2015; Tynes et al., 2015; Riehm et al., 2019; Plaisime et al., 2020; Maxie-Moreman and Tynes, 2022), a growing body of literature suggests there may be positive developmental outcomes associated with Black youths’ social media use. Some studies point to social media use as a tool in positive identity processes for Black youth, including the development of both *racial identity* (Harlow and Benbrook, 2019; Rogers et al., 2021b) and *science, technology, engineering, and mathematics identity* (STEM identity; Steinke, 2017; Nguyen and Riegle-Crumb, 2021). Racial identity (e.g., thoughts, feelings, and actions associated with racial group membership) has been shown to function as a protective and promotive factor (Sellers et al., 1998; Fergus and Zimmerman, 2005; Neblett et al., 2012) and is linked to positive indicators of health and academic achievement for Black youth (Jones and Neblett, 2016; Miller-Cotto and Byrnes, 2016; Loyd and Williams, 2017). Similarly, STEM identity (the extent to which one feels like a STEM person) is linked to an increased likelihood of pursuing a STEM major and engaged learning in STEM (Dou et al., 2019; Dou and Cian, 2022). Further, although distinct processes, there is evidence to suggest that racial identity may serve as a protective and promotive factor linked to Black youths’ persistence in STEM (Morton et al., 2019; Ortiz et al., 2020), emphasizing the need to understand how these identity processes unfold concurrently over time. Though there is an extensive literature on the processes that drive positive identity outcomes in *offline* contexts (e.g., peer relationships in schools; Rivas-Drake et al., 2017; Dou et al., 2019), it is less clear how Black youth navigate social media contexts as a space for dreaming and positive identity development.

To guide future investigations in this area, we present a conceptual model on the linkages between social media use, racial identity, and STEM identity development among Black youth. We begin by reviewing two frameworks that provide the theoretical foundations for our model: *positive youth development* (PYD; Lerner et al., 2005) and the *phenomenological variant of ecological systems theory* (PVEST; Spencer et al., 1997; Spencer, 2006). We then discuss our outcomes of interest: racial identity and STEM identity. Given its rapidly evolving nature, we then proceed with a discussion about how social media may be operationalized, noting limitations in the current literature, and highlighting the unique ways in which social media differs from offline contexts. Subsequently, we present our conceptual model, which we situate within distal- (e.g., digital redlining; Skinner et al., 2021), proximal- (e.g., access to mentors; Beauchamp et al., 2021), and individual-level (e.g., platform use motivation; Throuvala et al., 2019) offline influences. We also propose processes that may link social media use to positive identity outcomes and conclude with recommendations for future investigations.

## Theoretical foundations

Our conceptual model is built upon two premises. The first is that social media contains contextual resources that Black youth can access and leverage to facilitate positive identity development. The second is that Black youth are active agents in shaping their social media landscapes by engaging social-cognitive and behavioral strategies to resist disempowering messages and maximize affirming experiences. Here, we discuss two theoretical frameworks to substantiate these premises: positive youth development (PYD; Lerner et al., 2005) and the phenomenological variant of ecological systems theory (PVEST; Spencer et al., 1997; Spencer, 2006).

### Positive youth development

PYD builds upon the theoretical foundations of developmental systems theories by emphasizing that positive developmental trajectories for youth are driven by the alignment of individual assets with contextual supports (Lerner et al., 2005; Benson et al., 2012). Supportive relationships, particularly between youth and caring adults, are thought to bridge these individual and contextual factors and function as a scaffold for positive youth development outcomes (Bowers et al., 2015). Positive social feedback, often embedded in supportive relationships, supports positive identity development by signaling acceptance of youth’s authentic self (Harter, 1999; Shernoff, 2013). The development of positive racial and STEM identities has implications for Black youths’ well-being and persistence in STEM (Miller-Cotto and Byrnes, 2016; Dou et al., 2019). Thus, for Black youth, relationships with adults and peers who nurture and affirm their racial and STEM identities are a critical resource for positive youth development. The potential for supportive adults to foster positive racial and STEM identity development among racially diverse youth has been documented among out-of-school time (Loyd and Williams, 2017) and STEM programs (Price et al., 2019). We argue that social media, though fraught with risk factors, also contains tools and resources that expand access to affirming relational networks and supportive social feedback that may similarly drive positive identity formation for Black youth (Williams, 2019).

Scholars have similarly advocated for the application of PYD perspectives to understand how youth develop within social media contexts (see Ross and Tolan, 2021). PYD perspectives offer a useful theoretical frame for researchers to broadly understand the potential for positive identity development when Black youth are connected to affirming contextual resources (e.g., supportive relationships) on social media. However, overreliance on PYD perspectives may limit the field’s understanding of how Black youth cope with, navigate, and resist non-affirming social media experiences in the broader context of societal inequities. Specifically, PYD has been criticized for not adequately addressing the role of race and ethnicity (Williams and Deutsch, 2016) and structural oppression (Ginwright and James, 2002) in shaping access to opportunities for youth of color. Black youth face racism-related threats in multiple contexts across the life course (Jones et al., 2020; Seaton, 2020), and these threats manifest on social media in ways that are both interpersonal (e.g., online racial discrimination; Tynes et al., 2020) and structural (algorithm bias; Angwin et al., 2017; Peterson-Salahuddin, 2022). For example, algorithm bias on social media platforms has been shown to suppress the visibility of content from Black users (Peterson-Salahuddin, 2022). This threatens Black youths’ ability to engage positively with race-and STEM-related content on social media. Additional frameworks are needed to account for the strategies Black youth employ to resist racism-related experiences and maximize affirming experiences on social media to achieve positive identity outcomes.

### Phenomenological variant of ecological systems theory

The phenomenological variant of ecological systems theory (PVEST; Spencer et al., 1997; Spencer, 2006) compliments PYD perspectives by accounting for the roles of identity and oppression. PVEST emphasizes the importance of phenomenology (the social-cognitive process of meaning-making) for marginalized youth as they navigate, reflect on, and interpret their experiences with oppression in different social contexts over the course of development. In addition to dealing with common developmental stressors that all youth experience, Black youth are burdened with formulating coping strategies in the face of chronic, racism-related experiences that signal that they (and their dreams) are not valued across ecological contexts (Spencer et al., 1997; Seaton, 2020). This negative social feedback then triggers psychological defense mechanisms so that Black youth can continue to see themselves as valued contributors to their social space (Rogers, 2018; Jones et al., 2020). Spencer et al. (1997) note that the meaning-making processes that Black youth engage in response to racism-related stress informs the coping strategies they employ to ameliorate this distress. Racial identity formation is an inherently phenomenological process for Black youth (Velez and Spencer, 2018). As they learn more about the historical, sociocultural, political, and economic dimensions around what it means to be Black in the United States, they are concurrently developing a social-cognitive frame that informs their interpretation of past, present, and future race-related experiences, including the modes of support available to them. For instance, scholars have noted that the development of positive racial identity is linked to collective, behavioral strategies of resistance against racial injustices (e.g., civic engagement; Hope and Spencer, 2017). Thus, the PVEST framework emphasizes the importance of Black youths’ social-cognitive and behavioral strategies to attain support in light of racism-related stressors.

As Spencer et al. (1997) discuss, Black youths’ meaning-making around racism-related stressors and available coping supports may differentially lead to adaptive or maladaptive functioning over time. As discussed, social media is replete with risk factors and opportunities for negative experiences (Rose and Tynes, 2015; Tynes et al., 2015; Edwards et al., 2016; Riehm et al., 2019; Plaisime et al., 2020; Maxie-Moreman and Tynes, 2022). However, we advance the idea that social media also contains protective and promotive tools for Black youth and their identity development, such that it functions as a “high-risk, high-reward” context. Specifically, social media may expand Black youths’ perceived repertoire of support and facilitate positive development despite offline and online risks. Substantiating this notion, Evans (2022) used traditional and digital ethnographic methods to explore Black youths’ (ages 14–24) social media use while they participated in a hip-hop based education program in Chicago. Though participants faced multiple offline (e.g., neighborhood disadvantage, school exclusion) and online (e.g., algorithm bias; see Peterson-Salahuddin, 2022) risk factors, the author found that Black youth employed multifaceted strategies (e.g., collective posting, intentional hashtag use, joining shared-interest groups) to resist algorithm bias, increase visibility of their music content, and connect with shared-interest peers both online and offline. These social media strategies helped facilitate Black youths’ sense of connection to their racial and cultural identities while promoting their sense of efficacy as aspiring music professionals. These findings underscore how social media use, especially when combined with offline supports, can function as a promotive tool in Black youths’ meaning making and facilitate positive identity development. Although Evans (2022) focused on Black youths’ social media use in the context of hip hop education programming, we believe the emerging findings (e.g., collaborative social media use with same-interest peers and adults) have implications for STEM programming and Black youths’ racial and STEM identity development.

To summarize, PVEST complements PYD by considering Black youths’ social-cognitive and behavioral competencies in resisting oppressive forces and aligning ecological resources to achieve positive identity outcomes. In turn, identity functions as a psychosocial asset and cognitive frame that informs how Black youth experience, reflect on, navigate, and respond to online and offline social experiences.

## Identity development

Identity development, broadly, is a widely accepted developmental process for all youth, which involves crafting and understanding their place in the social world (Erikson, 1950). Across the life course, individuals’ sense of self, which involves thoughts, feelings, actions, and behaviors, becomes more differentiated and complex but integrated over time (McAdams, 2013). The ways Black youth engage with and are affirmed by the social environment allows them to establish an identity that affords adaptive functioning, through a perspective of development that incorporates multiple dimensions of diversity (e.g., race, ethnicity, gender, socioeconomic status, and interests; Spencer, 2006). Although many intersecting identity domains are important for individuals living in diverse historical, cultural, and political contexts (Galliher et al., 2017), in this article, we describe how this process may occur for two specific domains of Black youth’s identity: racial identity and STEM identity.

### Racial identity

*Racial identity* is a critically important facet of Black youth’s identity development (Neblett et al., 2012; Williams et al., 2020). For Black youth, racial identity involves the *process* through which they explore, interrogate, negotiate, and come to understand the meaning behind their racial group (what does it mean to be Black?). Further, it involves the *content* of the meaning they derive that may include thoughts, attitudes, and feelings (what do I think and how do I feel about being Black?). Inevitably, this process also involves Black youth attempting to reconcile their thoughts and feelings based on their observations and experiences in society and in historical context (Spencer, 2006; Brittian, 2012). To date, most research suggests that racial identity is generally linked to positive developmental outcomes in Black youth, including higher self-esteem, academic achievement, and mental and behavioral health (Neblett et al., 2012; Umaña-Taylor et al., 2014; Yip, 2018). Since the goal of this paper is to position social media as a place for Black youth to dream, we focus on the potential for positive racial identity development, although we acknowledge the potential for risk to exist as well (e.g., Tynes et al., 2020; Tao and Fisher, 2022).

Presently, the literature regarding racial identity considers this aspect of identity to be complex and multi-dimensional (Lee and Ahn, 2013; Umaña-Taylor et al., 2014) and a facet of development that begins early for Black youth (e.g., Murray and Mandara, 2002). Moreover, the variability in process differentially links to indicators of well-being. For example, cognitive and behavioral actions such as exploration and interrogation do not conclusively or automatically lead to positive feelings (also described as affect) or meaning making (Rogers and Way, 2018; Rogers et al., 2021a). In some cases, exploration may lead Black youth to discovering issues of historical and contemporary injustice (Mims and Williams, 2020). However, exploration can also lead to critical reflection about social inequities and for some Black youth become a catalyst for action and activism (Hope et al., 2019; Smith and Hope, 2020). Additionally, researchers have identified that variations in content also differentially relate to well-being. For example, beliefs and feelings around racial pride and affirmation are typically linked to positive development (Rivas-Drake et al., 2014). Some studies have identified negative beliefs and feelings around internalized shame can also be associated with racial identity development (due to racism-related experiences), which can consequently hinder or undermine other indicators of positive development (Johnson, 2020). While we recognize that social media has the potential to reinforce these negative evaluations through the proliferation of negative portrayals of Black people (Adams-Bass et al., 2014; Sullivan and Platenburg, 2017), our conceptual model explicates the ways in which social media also affords Black youth expanded access to a range of positive and affirming race-related content.

### STEM identity

What does it mean to develop a STEM *identity*? Scholars have demonstrated that STEM identity development begins in childhood and continues through adolescence (Hachey, 2020). Children begin to formulate concepts about *what* STEM careers entail and *who* can be a STEM professional based on their early STEM-related experiences (Dou et al., 2019). Broadly, STEM identity has been conceptualized as having at least two components: STEM-related *skills* and sense of STEM *community*. Expectancy-value theory (Eccles et al., 1983; Eccles, 2009) situates STEM skill development within cognitive, motivational, and social processes. According to this perspective, youth develop appraisals of their STEM-related skills (e.g., mathematics, analytical reasoning; Siekmann and Korbel, 2016) and, consequently, expectancies around their ability to conduct STEM-related tasks. These expectancies are informed by social feedback received during educational experiences. The desire to further develop these skills is also motivated, in part, by the perceived value of these skills in achieving intrinsic (e.g., finding STEM fulfilling; Boekeloo et al., 2015) and/or extrinsic (e.g., STEM leading to good-paying jobs; Hossain and Robinson, 2012) goals.

STEM identity has also been conceptualized as developing within communities of practice. Wenger (1998) defines communities of practice as social networks that endeavor to build and refine practitioners with a repertoire of skills in service of shared goals and values. Communities of practice perspectives foreground the role of mentorship as a social process by which skills and knowledge are developed and transmitted (Kezar et al., 2017). Notedly, access to mentorship has been identified as a critical resource associated with Black youths’ sense of efficacy and belonging in STEM (Martin, 2000; Brittian et al., 2009; Davis et al., 2019).

Two theoretical models have successfully synthesized and extended the skills and community dimensions of STEM identity in the context of Black youths’ identity development: Content Learning and Identity Construction (CLIC; Varelas et al., 2012) and Black Student STEM Identity (BSSI; Collins, 2018). CLIC posits that Black youths’ STEM identity develops through the ongoing process of meaning making, attending to both the content of the STEM community (e.g., tools, language, skills) and their relationships within the STEM community (e.g., connections to others in STEM; Varelas et al., 2012). BSSI foregrounds how contextual assets (e.g., mentors) and barriers (e.g., underrepresentation) differentially impact Black students’ persistence in STEM as they attempt to integrate their racial and STEM identities (Collins, 2018). Both models emphasize how racial and STEM identities develop concurrently and bidirectionally among Black youth. We expand on this in our own conceptual model, advancing the idea that social media may serve as a resource in which Black youth can build their STEM skills, expand their sense of STEM community, and facilitate integration of their racial and STEM identities.

## Conceptualizing social media

Over the past two decades, social media platforms have evolved rapidly. Compared to prior decades, Black youth now have access to a menu of social media platforms that have become increasingly interconnected and offer an array of content modalities that can be exchanged with remarkable speed. Though many social media platforms share similarities in this regard, Aichner et al. (2021) note that differences exist between platforms along several dimensions, including platform structure, primary content modality, target audience (s), and user motivation (s) for using certain platforms. Understandably, conceptualizations of social media use in the psychological literature have struggled to keep pace with the breadth and variability of social media use. For example, scholars have tended to operationalize social media use as a dose-effect construct based on frequency (e.g., time spent on social media per day; Riehm et al., 2019) or have focused on user experiences within a single platform (e.g., Black users’ Twitter experiences; Harlow and Benbrook, 2019). While these operationalizations confer methodological benefits to their respective studies, they obfuscate user-level (e.g., frequencies, motivations) and platform-level (e.g., interface, algorithm) differences *between* social media platforms. In turn, variability in user-and platform-related aspects may be differentially associated with identity development processes for Black youth. Thus, we present a conceptualization of social media that attempts to account for the breadth and variety of online tools available to them.

Similarly recognizing the difficulty of conceptualizing social media, Carr and Hayes (2015) offer the following definition:

Social media are Internet-based channels that allow users to opportunistically interact and selectively self-present, either in real-time or asynchronously, with both broad and narrow audiences who derive value from user-generated content and the perception of interaction with others. (p. 50)

This definition contains three conceptual points that are worth noting. First, it emphasizes the interactive and dynamic nature of social media. Traditional forms of media (e.g., film, television) have been characterized by a unidirectional flow of information from creators to consumers (Van Dijck and Poell, 2013). Notedly, this unidirectional dynamic is also present in teacher-centered approaches to K-12 learning where student input is minimal (Serin, 2018). Thus, in contrast with both traditional media and educational contexts, social media offers users the opportunity to be both creators *and* consumers of content. Hypothetically, this feature has the potential to position Black youth as active and engaged agents who can dynamically and collectively shape the content and relational networks embedded in their social media contexts (Spencer, 2006; Halpern, 2017).

Second, it alludes to the role of anonymity (i.e., “selectively self-present”) on social media platforms. Compared to in-person contexts, social media users are generally afforded more discretion in deciding the breadth and depth of personal information they would like to share with others (i.e., self-disclosure; Misoch, 2015). This aspect of social media may confer benefits to content exploration in social media contexts. Whereas participation in in-person activities tends to be readily observable by others (e.g., attending an afterschool science program), participating in activities on social media (e.g., viewing science content) is more difficult for outsiders to observe. The former raises the prospect of receiving social feedback which, in some cases, may not be desired. Thus, anonymity on social media may serve a protective function among users who are interested in exploring certain content while modulating unwanted social feedback.

Third, the definition by Carr and Hayes (2015) acknowledges that multiple audiences are embedded in social media contexts. Offline, Black youth are developing and navigating social interactions within multiple ecological contexts (e.g., families, schools, neighborhoods; Spencer, 2006; Bronfenbrenner and Morris, 2007). The increasing globalization of social media offers Black youth opportunities to connect with multiple social groups beyond their offline contexts. Although this aspect of social media has the potential to confer risks to Black youth with the prospect of hostile social interactions (e.g., experiencing online racial discrimination; Tynes et al., 2020), it may also connect them to supportive groups and positive experiences, particularly around shared cultural experiences. Notedly, this may include exposure to the content of Black STEM professionals, which has implications for Black youths’ racial and STEM identity development (Martin, 2000; Varelas et al., 2012; Brown et al., 2017; Collins, 2018; White et al., 2019). We expound upon this point in our conceptual model.

We augment this conceptualization of social media by Carr and Hayes (2015) by offering an additional dimension to consider: *content intrusiveness*. Derived from existing literature on social media advertising (Huang, 2019; Noguti and Waller, 2020), we define content intrusiveness as the degree to which users are exposed to content on a social media platform in a way that is unsolicited and not as a direct result of information-seeking behaviors. Said differently, it is the degree to which exposure to content on social media is driven primarily by the platform (i.e., high content intrusiveness) rather than the user (i.e., low content intrusiveness). Social media platforms that are high in content intrusiveness may have the potential to expose Black youth to both positive and negative messages related to their identity and interests. This may function as a “high-risk, high-reward” context for Black youth in the course of identity development as they navigate content that may contain novel identity-related information but may differ in socio-emotional valence (i.e., the extent to which content elicits positive or negative feelings; Michikyan et al., 2014).

In summary, current conceptualizations of social media in the psychological literature often obscure between-platform differences which, in turn, limits our ability to account for the tools available to Black youth for identity development processes. Compared to traditional media and offline contexts, social media platforms offer Black youth unique tools such as content creation, anonymity, and expanded access to diverse social groups. Further, content intrusiveness varies between platforms, which has implications for how Black youth are exposed to social media content and the different competencies they employ on each platform.

## Conceptual model

**Figure 1:**
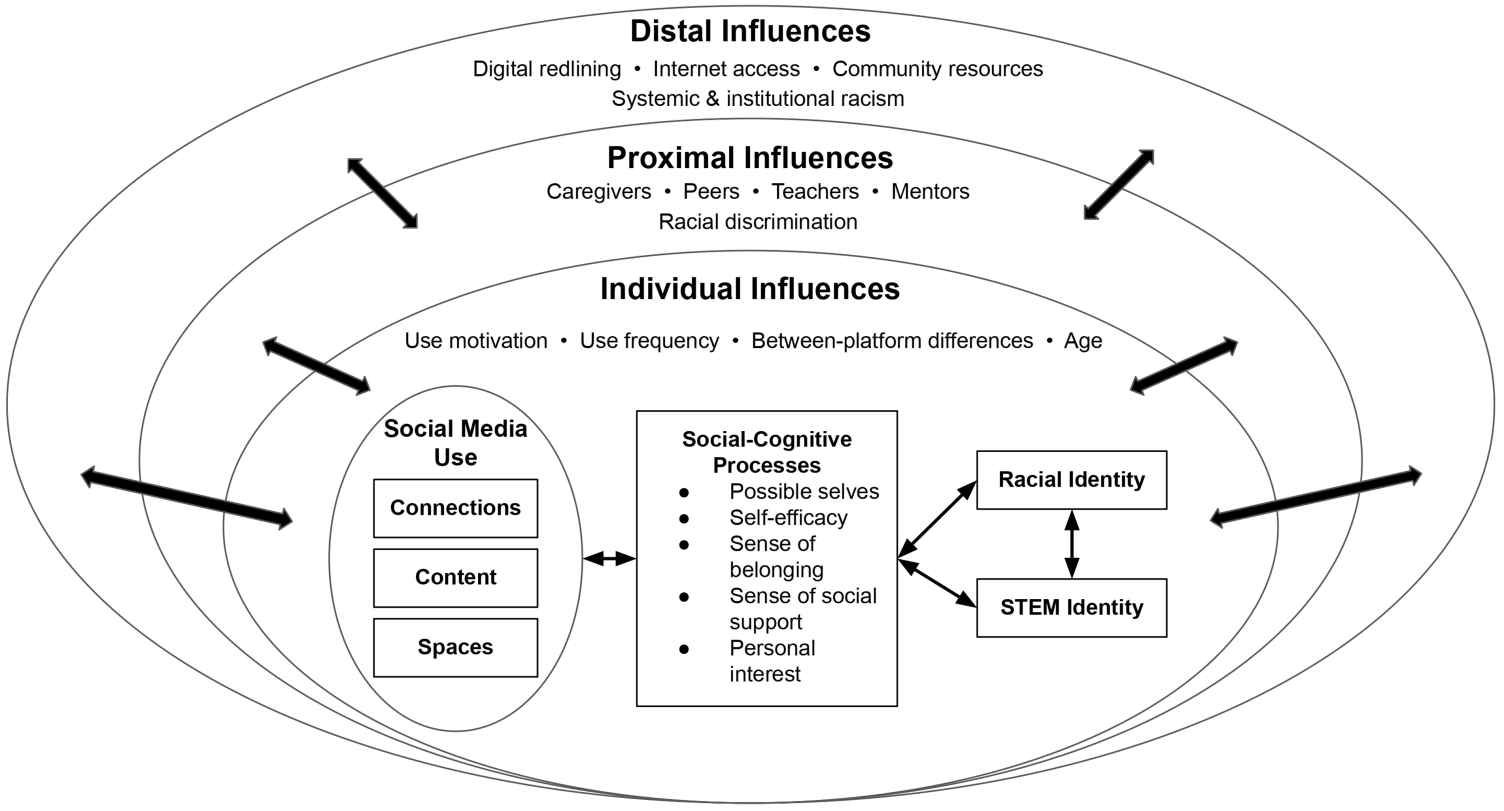
Conceptual model illustrating associations between offline influences, social media use, and racial and STEM identity outcomes.

In this section, we elucidate components of our conceptual model (Figure 1) as they relate to the development of racial identity and STEM identity in Black youth. We recognize this process as iterative and cyclical with changes in identity outcomes interactively influencing subsequent online and offline experiences in the context of racism-related stressors (Spencer, 2006). Multiple disciplines have examined offline influences on identity development long before the advent of contemporary social media platforms. These offline processes serve as both antecedents to and ongoing influences on Black youths’ social media use. Here, we briefly acknowledge some of these influences at the distal (i.e., contexts furthest from the youth), proximal (i.e., contexts closest to the youth), and individual (i.e., within and between youth) level.

### Distal influences

Black youths’ social media use and identity development occur within a nexus of sociopolitical and economic structures that moderate their access to the Internet and other community resources. Internet access is facilitated by public investment in broadband infrastructure (Cambini and Jiang, 2009). Due to the intergenerational effects of redlining (Aaronson et al., 2021), Black youth may disproportionately reside in neighborhoods in which broadband infrastructure is poor or inaccessible. In a compelling analysis, Skinner et al. (2021) found that in-home broadband access decreased among urban and suburban neighborhoods that had been historically redlined by the U.S. government. Rural communities of color face even greater deficits in broadband investment (Friedline et al., 2020). The inequitable distribution of broadband infrastructure and Internet access is sometimes referred to as “digital redlining” (Skinner et al., 2021). Despite these barriers, Black youth may be adapting to digital redlining by accessing social media platforms in clever ways, such as through device sharing (Matthews et al., 2016) or by visiting public libraries (Hall, 2021). As interest in Black youths’ social media use grows, scholars and educators should be mindful that these adaptive strategies may be formulated *in response to* historically rooted disinvestment in Black communities. Future studies should continue to consider the role of structural inequities on social media use and broader implications for Black youths’ health, development, and adaptive functioning.

Whereas digital redlining presents a distal barrier to Black youth, other distal influences may enable access to race-and STEM-related resources in offline contexts. For example, public and private organizations can offer Black youth increased opportunities for racial identity exploration, such as through cultural events (e.g., festivals, celebrations), Black-owned businesses, and African American art and history museums. Access to racially affirming community spaces has been shown to play a promotive role in Black youths’ racial identity development (Loyd and Williams, 2017; Mroczkowski et al., 2021). Relatedly, organizations can provide Black youth with STEM experiences through school-or community-based programming. Organizations such as *Girls Who Code* and the *National Society for Black Engineers* (both of which have thousands of local chapters throughout the United States) endeavor to provide culturally competent STEM mentorship to promote the retention of Black youth in the STEM pipeline. These offline resources represent opportunities for Black youth to access supportive adults and racially affirming communities of practice (Varelas et al., 2012; Bowers et al., 2015; Kezar et al., 2017). Both school-and community-based STEM programs have evidenced the potential to promote STEM identity development among Black youth (Martin, 2000; Loyd and Williams, 2017; Tan and Barton, 2018; Price et al., 2019). Thus, distal influences operate differentially to facilitate or obstruct opportunities for Black youths’ racial and STEM identity development. Additionally, Black youth may access information about distal resources through social media.

### Proximal influences

Relationships at the proximal level help bridge connections to distal resources (or overcome distal barriers) for Black youth. It is widely acknowledged that Black youths’ identity development unfolds within meaningful relationships, including relationships with caregivers, peers, teachers, and mentors. The constellation of social processes embedded in these relationships, and their potential influence on Black youths’ identity outcomes, are rich and nuanced (Lesane-Brown, 2006; Hughes et al., 2011, 2016; Smetana et al., 2015). We briefly highlight some of the identity-promoting processes that may occur in these relationships, starting with caregivers.

Through the process of racial socialization, Black caregivers (e.g., parents, grandparents) transmit a range of messages, values, and information about racial group membership to their children (Peters, 2002; Hughes et al., 2006). Racial socialization, in the context of a supportive caregiver-youth relationship, has the potential to shape positive trajectories in Black youths’ racial identity development (e.g., enhancing private regard; Williams and Smalls-Glover, 2014). Black caregivers also equip their children with cultural coping skills (e.g., preparation for bias; Hughes et al., 2009; Anderson et al., 2020; Scott et al., 2020) to help them resist the detrimental effects of discrimination and unfair treatment. Similarly, caregivers facilitate the growth of Black youths’ STEM identity by providing instrumental support (e.g., furnishing transportation to STEM programs; King et al., 2021), emotional support (e.g., affirming feedback on STEM interests; Park-Taylor et al., 2022), and access to social capital (e.g., STEM professionals in the family network; Saw, 2020).

Peer relationships become increasingly important during adolescent development (Brown and Larson, 2009). For Black youth, peer relationships represent a potential resource for positive racial and STEM identity development. The availability of Black peers, particularly in school contexts, is associated with higher racial identity exploration and affirmation among Black youth (Derlan and Umaña-Taylor, 2015). These relationships are also associated with increased racial centrality (i.e., importance of racial identity to the self), which may serve a protective function in school contexts in which same-race peer availability is low (Douglass et al., 2017; Santos et al., 2017; Hoffman et al., 2019). Similarly, the promotive role of peers has been documented for STEM identity development. Dou et al. (2019) found that children who talked about STEM-related topics more frequently with their peers were more likely to report a stronger STEM identity in high school, suggesting that peer dynamics feature prominently in youths’ STEM identity development. Notedly, emotional and instrumental support provided by same-race peers is associated with Black youths’ feelings of school belonging (Tatum, 2004), academic achievement (Byrd and Chavous, 2011), and STEM persistence (Lancaster and Xu, 2017).

Teachers play a powerful role in Black youths’ racial and STEM identity development by shaping their sense of belonging in the classroom. Black students disproportionately face discriminatory and exclusionary treatment from their teachers (e.g., tracking and teacher bias; Lucas and Berends, 2007), which threatens positive racial and STEM identity development. However, scholars have also identified characteristics of the student-teacher relationship that may be promotive for Black youth. Legette et al. (2020) persuasively illustrate that a healthy student-teacher relationship is predicated on the teacher recognizing the humanity of their Black students. One way that teachers may convey this message is by signaling that Black youths’ racial identity is valued in the classroom, such as through the utilization of culturally sustaining curricula and race-affirming messages (Ladson-Billings, 2014; Quinn, 2020). Recognizing the value Black youth bring to the classroom also undergirds the strategies teachers can employ to nurture Black youths’ STEM identity. Teachers may do this by taking the time to develop Black youths’ STEM-related skills (e.g., science, mathematics; Richards and Robertson, 2016), recruiting and supporting them through advanced coursework (Davis et al., 2019; Grissom et al., 2020), sharing information about paths to different careers in STEM (Craig et al., 2019), and affirming their brilliance in STEM (Gholson and Robinson, 2019). Thus, when teachers celebrate the contributions of their Black students, and take the time to nurture their academic development, this has the potential to contribute positively to Black youths’ racial and STEM identity trajectories.

Mentors (i.e., supportive nonparental adults) also feature in Black youths’ identity development. Similar to caregivers and teachers, mentors may provide emotional and instrumental support to Black youth in ways that nurture racial *and* STEM identity development. Substantiating this point, Hurd et al. (2012) found that Black youth who reported having support from a mentor also reported higher private regard towards their racial identity and exhibited more positive long-term educational attainment. A close mentor-mentee relationship may be an important context in which mentors transmit social and cultural skills to Black youth which, in turn, enhances Black youths’ social and educational competencies (Hurd and Sellers, 2013). Importantly, Black youth have identified access to mentors as integral to their persistence in STEM in previous studies (Martin, 2000; Brittian et al., 2009; Davis et al., 2019; Beauchamp et al., 2021). Notably, having access to same-race mentors in STEM may facilitate racial and STEM identity integration (see Collins, 2018) by expanding Black youths’ range of possible selves (Oyserman and James, 2009; Burt and Johnson, 2018; Wade-Jaimes et al., 2021).

### Individual influences

Black youths’ social media use is influenced by individual-level interests and motivations that are developed within the distal and proximal influences previously discussed. Namely, Black youth may have differing motivations for using social media in ways that inform which platforms they use and how they use them. For instance, they may use platforms with a more user-driven experience to maintain offline relationships (e.g., Snapchat, Facebook; Butler and Matook, 2015; Throuvala et al., 2019) or platforms higher in content intrusiveness to explore new content and connections (e.g., Twitter, TikTok; Harlow and Benbrook, 2019; Klug et al., 2021). Further, a desire to modulate social feedback by remaining anonymous might inform how Black youth navigate social media spaces (e.g., “lurking”; Edelmann, 2013). Lastly, Black youths’ social media use frequencies and motivations may differ between platforms. These “use profiles” may be associated with different processes through which Black youth engage with identity-related content (Scott et al., 2017).

### Social media use

As discussed previously, when Black youth use social media, they are entering an online context with unique features compared to their offline contexts. These features include bidirectional flow of information (i.e., content creation and consumption), the option of anonymity, expanded access to social groups, and content intrusiveness. Further, the preceding discussion foregrounds the importance of two aspects in Black youths’ identity development trajectories: (1) supportive relationships with caring adults and peers, and (2) access to racial-and STEM-promoting contextual resources. We posit that Black youth may use the tools and artifacts available on social media to enhance these aspects of positive identity development by maintaining offline relationships, exploring new content and connections, and trying different modes of self-representation.

Before the advent of Internet-based technologies, the potential for Black youth to benefit from supportive offline relationships would have been contingent on their ability to connect in-person. If a close friend, family member, or mentor moved away, the ability to maintain the connection (and benefit from it) would have been limited. With contemporary social media platforms, Black youth can maintain and enhance these offline connections in dynamic ways. For example, Facebook allows users to create private groups (i.e., users must be invited to the space to interact with other users; Rothschild and Aharony, 2021). Black youth can create or enter these spaces to maintain contact with multiple family members or peers that they trust. Further, users can exchange, engage with, and comment on a wide range of text and audiovisual content in these spaces. This facilitates opportunities for Black youth to discuss race-and STEM-related content with supportive adults and peers that they know, but may not have direct access to, in their offline contexts. Of note, private social media spaces with trusted offline users may function as a space in which Black youth can receive positive feedback about their STEM interests and skills. This may be particularly beneficial if they are situated in educational contexts marked by exclusionary practices.

In addition to maintaining offline relationships, we posit that social media expands and offers additional opportunities for Black youth’s racial identity exploration *via* access to new content and social connections. Based on prior literature, this may occur in two ways: as a context-driven process (e.g., racial socialization and exposure; Hughes et al., 2006; Neblett et al., 2009) and/or a youth-driven process (e.g., through youth’s interest and agency; Umaña-Taylor et al., 2013). In the former, social media might implicitly and explicitly expose Black youth to cultural content and information (e.g., historical and cultural knowledge, art, and music) that is not readily available in their immediate offline contexts, which can also facilitate racial pride and affirmation. For example, *Young Chicago Authors* is a non-profit organization that educates young people in the art of creative writing and the power of self-expression through workshops, events, and education. This organization has hosted spoken word events on Instagram. Representing mostly Black and Latinx youth and young adults, we believe that implicit and explicit racial socialization is likely occurring in these spaces. In the latter, as a youth-driven process, Black youth may also actively search for content that they find interesting and that speaks positively to their racial identity.

Similarly, access to racially affirming contextual support, particularly outside of schools, has the potential to keep Black youths’ STEM dreams alive (Davis et al., 2019; Price et al., 2019; Young et al., 2019; Tan and Barton, 2020). We believe that social media has the potential to enhance and expand these contextual supports for Black youth by connecting them to racially affirming STEM communities. For example, the *National Society for Black Engineers* (NSBE) has a prominent social media presence on Instagram and Twitter. In these digital spaces, Auguste et al. (2018) found that users exchanged instrumental support (e.g., resume drafting, study tips), emotional support (e.g., cultural affirmations), and professional opportunities (e.g., college preparation, job openings). Thus, social media has the potential to facilitate STEM identity development for Black youth by providing access to positive feedback and social support that may be limited in offline contexts. Relatedly, social media may expand access to the programming efforts of offline youth STEM organizations. For example, *YOUmedia* operates as a digital extension of the Chicago Public Library and endeavors to serve as a space to connect youth with books, media, and skilled staff for STEM and academic development. Among Black youth who have access to this space, this facilitates opportunities to develop STEM-related skills and learn about different STEM fields (e.g., graphic design, 2D/3D design, etc.).

Another unique facet of social media as it relates to Black youth’s identity development is the opportunity to create and alter one’s representation more easily relative to one’s offline representation. Being able to modify one’s visual representations (e.g., avatars, thumbnails; Subrahmanyam and Šmahel, 2011) offers Black youth the opportunity to enhance, reinforce, reflect, represent, or display aspects of their appearance that speak to their positive racial identity. In this regard, programmers and developers should be sensitive to offering a variety of options that allow Black youth to freely explore and create a representation of themselves in virtual space, such as modifiable skin color, facial features, and hair textures and styles. The representations that Black youth create may or may not exactly replicate or reflect youth’s offline physical appearance; exploration is indeed a normative process.

In summary, though social media platforms represent a place for negative experiences to occur, they also offer Black youth the opportunity to both seek out and create culturally affirming spaces. This may confer several benefits if they are situated in disempowering offline contexts (classrooms, schools, neighborhoods). Whether the process occurs through socialization or as a youth-driven process, we believe exposure may be particularly important for Black youth who are underrepresented in their schools or neighborhoods or for youth who do not readily have access to cultural or STEM information and role models. Importantly, culturally affirming spaces can also reinforce and celebrate Black youth for who they are and how they contribute to the collective space.

### Social-cognitive processes

Thus far, we have advanced the notion that social media may serve to expand access to race-and STEM-affirming resources for Black youth and have implicated the role of social-cognition (e.g., meaning-making) in the identity development process (Spencer, 2006; Velez and Spencer, 2018). We propose five social-cognitive processes that may mediate or influence the associations between social media use and positive identity outcomes: possible selves, self-efficacy, sense of belonging, sense of social support, and personal interest.

#### Possible selves

Before the explosion of social media use, identity scholars acknowledged the role of possible selves in identity development. Possible selves (i.e., versions of the self that people may want to become in the near and distant future; Oyserman and James, 2009) serve self-regulatory functions by governing how one directs their cognitive, affective, behavioral, and social resources to bridge the discrepancies between their current self and desired future self (Oyserman and Destin, 2010; Frazier et al., 2021). From a young age, Black youth contend with negative messages about their race through exposure to negative media portrayals and discriminatory treatment (Adams-Bass et al., 2014; Seaton, 2020). Black children similarly attend to representations of STEM early in development and begin to discern *who* is represented in STEM professions (Aladé et al., 2020; London et al., 2021). Black Americans continue to be underrepresented in the STEM workforce (Rivers, 2017; Graf et al., 2018), which limits the visibility of Black STEM representatives in Black youths’ offline contexts. Consequently, Black youth may struggle to craft a possible self in STEM when they do not see themselves reflected in these professions.

Social media may help ameliorate these barriers by connecting Black youth to cultural artifacts (e.g., hashtags) that expand the repertoire of possible selves (Gevisa, 2020). For example, social media campaigns such as “I, Too, Am” and @BlackInNeuro have increased the visibility of Black STEM professionals (Butler, 2014; George Mwangi et al., 2018; Diep, 2020; Murray et al., 2021; Carr et al., 2022). Although conducted with adults, we posit that these studies illustrate the potential to expand the range of possible selves for Black youth by mitigating negative racial stereotypes and signaling who gets to become a STEM person (Ginwright, 2007; Oyserman and James, 2009; Collins, 2018; Schlegel et al., 2019). It should be noted that while the tools of social media (e.g., selective self-disclosure) have the potential to proliferate unrealistic portrayals (e.g., perfectionism; Harren et al., 2021), these same tools confer Black youth increased agency to author positive identities and counternarratives against negative stereotypes (Rogers and Way, 2018; Rogers et al., 2021b). We believe when Black youth can see it, they can *dream* it.

#### Self-efficacy

Black youth may strive for a desired future self to the extent that they view the path to get there as feasible and attainable, implicating the role of self-efficacy. Self-efficacy, or one’s perception of their ability to achieve goals and effect change (Bandura, 2000; Eccles, 2009), is a prominent feature in theoretical and empirical work on Black youths’ racial and STEM identity development (Spencer, 2006; Varelas et al., 2012; Collins, 2018; White et al., 2019). Black youth face racism-related stressors in their educational and social contexts that present obstacles to positive identity development. Their ability to resist these negative social experiences (e.g., cultural coping; Anderson et al., 2019) and believe in their STEM-related abilities (Corneille et al., 2020) is undergirded, in part, by self-efficacy. In turn, developing self-efficacy is scaffolded by supportive social feedback and relationships (Martin, 2000; Brittian et al., 2009; Bowers et al., 2015; Davis et al., 2019). Social media may enhance Black youths’ self-efficacy by facilitating access to race-and STEM-promoting spaces. For example, digital learning spaces such as *YOUmedia* expand opportunities for Black youth to develop their self-efficacy in STEM by practicing skills and accessing supportive mentors and cultural artifacts. Similarly, the racially affirming social media spaces offered by organizations such as the *National Society for Black Engineers* may equip Black youth with a sense of efficacy in their abilities to meet the social and professional demands of STEM.

#### Sense of belonging

Belonging, or the feeling that one is valued in a social group, is widely regarded as a fundamental human need (Maslow, 1954; Allen et al., 2021). Identity development is intertwined with seeking belonging as youth gauge social feedback to determine which groups affirm their authentic selves (Spencer, 2006; Griffin et al., 2017). One aspect of Black youths’ racial and STEM identity development is connecting with adults and peers who affirm these facets of their identity (Gray et al., 2018). Social media represents opportunities for Black youth to connect with content and communities that value their racial and STEM identities. Of note, the increased visibility of racially affirming support on social media may bridge connections to offline support in higher education. This is a critical resource for Black youths’ persistence in STEM, particularly in predominantly White educational settings (Palmer et al., 2011; Morton et al., 2019). Interestingly, the sense of belonging cultivated through social media use may occur even when other users are unknown offline (Watkins et al., 2017) or under conditions of anonymity (De Choudhury and De, 2014).

#### Sense of social support

Engagement with affirming spaces and content on social media may enhance Black youths’ sense of social support. Social support has been conceptualized as consisting of emotional (e.g., offering comfort during emotional distress) and instrumental (e.g., helping with homework) dimensions (Taylor, 2011). Though social support and sense of belonging have conceptual overlap (Cohen and McKay, 1984), we posit that they operate as distinct processes in our model. Sense of belonging requires social interaction and connectedness with others. This may not be a prerequisite for social support in social media contexts, particularly for the dimension of instrumental support. For example, in the social media spaces described thus far, Black youth may be exposed to content that offers instructions (e.g., how-to videos) relevant to certain domains of racial identity (e.g., self-presentation, art, music) and STEM identity (e.g., application of scientific concepts, college preparation) development. The user who shares the instructional content may not be known to the youth. Nonetheless, this type of social media content may enhance Black youths’ sense of social support by equipping them with tools to meet desired goals (e.g., expression, achievement) within their racial and STEM identities.

#### Personal interest

The last process we propose, personal interest, is built on a simple premise: for Black youth to pursue STEM, they must be interested in and enjoy STEM. Enjoyment of STEM is broadly recognized as youths’ positive affect towards STEM tasks and activities (Falk et al., 2016; Burt and Johnson, 2018). Educators may facilitate children’s enjoyment of STEM early in development through the use of fun and active learning strategies (Bedard et al., 2019). As Black children transition into adolescence, gaining the ability to evaluate their social world more critically, enjoying STEM becomes increasingly tied to its perceived relevance to and reflection of their lived experience (Eccles et al., 1983; Kumar et al., 2018; Mark, 2018; Gray et al., 2020). The use of culturally relevant pedagogy in STEM education and programming is associated with Black youths’ increased interest, and persistence, in STEM (Mark, 2018; Gray et al., 2020; Younge et al., 2022). Engagement in racially affirming STEM spaces on social media may help facilitate feelings of enjoyment in STEM by linking its utility to Black youths’ sociocultural background and lived experiences.

### Intersecting racial and STEM identities

As suggested by the bidirectional arrow in our conceptual model, Black youths’ racial and STEM identity development are not mutually exclusive processes (Martin, 2000; Syed, 2010; Varelas et al., 2012; Brown et al., 2017; Collins, 2018; White et al., 2019). Rather, they are intertwined and contoured by historical, sociocultural, and economic forces. At the distal level, Black youth are disproportionately represented in under-resourced schools and neighborhoods, which constrains resources available for STEM skill development (e.g., access to technology; Lukes and Cleveland, 2021). Further, Black youth often contend with devaluing social messages through discriminatory and exclusionary practices (e.g., tracking, teacher bias, harsh discipline; Lucas and Berends, 2007; Kupchik and Ward, 2014; Blake et al., 2016). These practices threaten positive racial and STEM identity development by restricting access to social support (e.g., peers and teachers) and effectively signaling that Black youths’ STEM development is not valued in the education system. Over time, these negative social messages may contribute to STEM identity integration difficulties, particularly among high achieving Black youth, as they navigate the effects of negative stereotyping and differing attributions for their success in STEM (Larnell et al., 2014; Collins, 2018; Collins et al., 2020). Social media may offer Black youth a means of resisting these negative experiences, expand their opportunities to create and explore, and facilitate the integration of their racial and STEM identities (Syed and McLean, 2016). Future research might explore how these two domains of Black youth’s identities develop separately or in tandem over time (e.g., Syed, 2010). Aligned with PYD perspectives and PVEST, we suggest this dynamic process occurs iteratively within youth and bidirectionally between youth and their environment (online and offline) over the course of development.

## Discussion

Establishing a sense of identity is an important facet of development for all adolescents that involves crafting and creating, understanding, and negotiating their place in the social world (Erikson, 1950; Spencer, 2006). In this article, we focus on two prominent domains of identity for Black youth - racial identity and STEM identity - and present a conceptual model that elucidates how this complex and dynamic process may occur within youth and between youth and their environment. For Black youth, the process of establishing their identities around these two specific domains may involve resisting stereotypes and marginalization around race and STEM. At the same time, racial identity and STEM identity development can be a beautifully creative and affirming endeavor where Black youth forge a new and authentic path for themselves. Youths’ identity formation also occurs in the context of proximal influences, including youth’s relationships with family, peers, teachers, and mentors, and distal influences, including school, community resources, and access to technology. Notably, social media offers new opportunities for creation, influence, and engagement (Deaton, 2015; Kenna and Hensley, 2019; Dragseth, 2020). Just as in offline contexts, distal and proximal influences differentially constrain or support positive identity development that can occur through social media. We view social media as a “high-risk, high-reward” context, where proximal and distal influences intersect and where Black youth can engage intentional strategies to resist disempowering experiences and access tools to support their interests and development.

Investigations into how Black youth use social media to create and explore for identity development represents an important and exciting avenue of research. While there are several research questions scholars could pursue in this area, we suggest four based on our preceding discussion. First, what roles do passive (e.g., content exposure) and active (e.g., content creation) social media processes play in Black youths’ identity development? Second, what processes bridge offline and online experiences (e.g., caregiver conversations with youth)? Third, in what ways are Black youths’ offline and online identities congruent, and how might social media facilitate integration with other facets of identity (e.g., gender and sexual orientation)? Fourth, what role does social media play in Black youths’ development over the life course (e.g., from early adolescence to emerging adulthood)?

In conclusion, this special issue invites authors to consider the ways in which Black youth continue freedom dreaming. Both the online and offline world are replete with barriers and negative messages that threaten to minimize Black youths’ dreams and visions for the future. Yet, as a rapidly evolving and increasingly globalized forum, social media also affords Black youth unique tools and opportunities to resist barriers and cultivate social and educational experiences outside of their classrooms, schools, and neighborhoods. When Black youth are told to make their freedom dreams *smaller*, our hope is that future investigations will continue to reveal how Black youth use social media to make their freedom dreams *bigger* early and often.

## Author contributions

TL and AL developed the conceptual model, drafted the discussion section, and conducted final review and revisions. TL drafted the introduction, theoretical foundations, STEM identity, conceptualizing social media, and conceptual model sections. AL drafted the racial identity section. All authors contributed to the article and approved the submitted version.

## Funding

The preparation of this manuscript was supported by the Chancellor’s Distinguished Fellowship at the University of California, Riverside. AL’s work was supported in part by National Science Foundation award DRL# 1906954.

## Conflict of interest

The authors declare that the research was conducted in the absence of any commercial or financial relationships that could be construed as a potential conflict of interest.

## Publisher’s note

All claims expressed in this article are solely those of the authors and do not necessarily represent those of their affiliated organizations, or those of the publisher, the editors and the reviewers. Any product that may be evaluated in this article, or claim that may be made by its manufacturer, is not guaranteed or endorsed by the publisher.
